# Genetically driven predisposition leads to an unusually genomic unstable renal cell carcinoma

**DOI:** 10.1007/s12672-024-00894-5

**Published:** 2024-03-21

**Authors:** Manuel Scimeca, Valentina Rovella, Sabrina Caporali, Yufang Shi, Julia Bischof, Jonathan Woodsmith, Giuseppe Tisone, Giuseppe Sica, Ivano Amelio, Gerry Melino, Alessandro Mauriello, Pierluigi Bove

**Affiliations:** 1 Department of Experimental Medicine, TOR, University of Rome Tor Vergata, 00133 Rome, Italy; 2 Division for Systems Toxicology, Department of Biology, University of Konstanz, 78457 Konstanz, Germany; 3 The Third Affiliated Hospital of Soochow University, Institutes for Translational Medicine, Soochow University, Suzhou, 215000 China; 4 Indivumed GmbH, Falkenried, 88 Building D, 20251 Hamburg, Germany; 5 Department of Surgery, TOR, University of Rome Tor Vergata, 00133 Rome, Italy

## Abstract

Renal cell carcinoma originates from the lining of the proximal convoluted renal tubule and represents the most common type of kidney cancer. Risk factors and comorbidities might be associated to renal cell carcinoma, while a small fraction of 2–3% emerges from patients with predisposing cancer syndromes, typically associated to hereditary mutations in *VHL, folliculin, fumarate hydratase* or *MET* genes. Here, we report a case of renal cell carcinoma in patient with concurrent germline mutations in *BRCA1* and *RAD51* genes. This case displays an unusual high mutational burden and chromosomal aberrations compared to the typical profile of renal cell carcinoma. Mutational analysis on whole genome sequencing revealed an enrichment of the MMR2 mutational signature, which is indicative of impaired DNA repair capacity. Overall, the tumor displayed a profile of unusual high genomic instability which suggests a possible origin from germline predisposing mutations in the DNA repair genes BRCA1 and RAD51. While *BRCA1* and *RAD51* germline mutations are well-characterised in breast and ovarian cancer, their role in renal cell carcinoma is still largely unexplored. The genomic instability detected in this case of renal cell carcinoma, along with the presence of unusual mutations, might offer support to clinicians for the development of patient-tailored therapies.

## Introduction

Renal cell carcinoma (RCC) is the most common malignancy that arises from the kidney accounting for ~ 80% of kidney cancers and approximately for 3–5% of all tumours [1]. According to the last WHO [2], its classification requires a combination of morphological, molecular, and genetic characteristics. The major subtypes include clear cell (ccRCC), papillary (pRCC), and chromophobe (chRCC) RCC [2, 3], which originate from different segments of the nephron, either proximal (ccRCC, pRCC) or distal (chRCC). The main characteristics of the RCC are late diagnosis (due to the specific anatomical site), tendency to metastasize and a remarkable chemoresistance. Approximately 20–40% of patients with localized RCC experience disease recurrence after surgery. While therapeutic options have improved, particularly for ccRCC [4], the response in metastatic patients and 5-year survival rates remain unsatisfactory. Although clinicopathological scoring systems like the clinical International mRCC Database Consortium model [5] can stratify metastatic RCC patients regardless of their subtype [3, 5], significant differences in clinical outcomes are observed within each prognosis group.

RCC might manifest as hereditary forms, accounting for 2% of all renal neoplasia; mostly associated to germline mutations of Von Hippel–Lindau (VHL) and folliculin (*FLCN*) genes [6, 7]. Other autosomal dominant inherited syndromes associated to aggressive kidney cancers are hereditary Leiomyomatosis and Renal Cell Carcinoma (HLRCC) caused by loss of function of Fumarate Hydratase (FH), key enzyme of TCA cycle [8], Hereditary Papillary RCC (HPRC) which is linked to activating germline mutations in MET Proto-Oncogene tyrosine kinase receptor (*MET*) gene [9].

Over the years, analysis of cohort of familiar forms of RCCs pointed out pathogenic cancer-associated germline variants with unclear role in RCC pathogenesis [10, 11]. The genetic profile of RCC subtypes, including p53 [12–19] (see Table 1) might include PBRM1 mutations, that display upregulation of several genes involved in the angiogenesis, with increased response to VEGF-target therapy [20], VHL-deficiency in RCC is associated to a vascular development gene expression signature triggered by VHL/HIF pathway which can be target by HIF-2a inhibitors [21] while increased TH2 immune gene expression signature is strongly associated to poor prognosis and lower survival [22].

**Table 1:** Most common gene alterations found in RCC subtypes

Genes	Function	RCC subtype
*VHL*	Ubiquitination/degradation of hypoxia-inducible-factor (HIF)	ccRCC
*PBRM1, ARID1, SMARCA4*	Chromatin remodeling SWI/SNF complex	ccRCC
*BAP1*	Polycomb Repressive Deubiquitinase complex (PR-DUB)	ccRCC
*SETD2*	Histone methyltransferase (H3K36me3)	ccRCC
*EZH2*	Polycomb Repressive complex 2 (PRC2)	ccRCC
*MLH1, MSH2, MSH6, PMS2*	Mismatch Repair	ccRCC
*MET*	MET Proto-Oncogene tyrosine kinase receptor	pRCC
*TERT*	Telomerase reverse transcriptase	pRCC
*CDKN2A*	Cyclin dependent kinase inhibitor 2A	pRCC
*CDKN2B*	Cyclin dependent kinase inhibitor 2B	pRCC
*EGFR*	Epidermal Growth factor receptor	pRCC
*TP53*	Tumor suppressor protein p53	chRCC
*PTEN*	Tumor suppressor Phosphatase and tensin homolog	chRCC

See for details references [20, 21]

Moreover, understanding the RCC microenvironment has opened new therapeutic strategies with immune check point inhibitors as anti- cytotoxic T-Lymphocyte Antigen 4 (anti-CTL4) and anti-programmed cell death-1 (anti-PD-1) monoclonal antibodies [23]. In particular, the anti-PD-1 treatment have shown strong clinical benefit in renal cell carcinomas characterized by deficiency in genes of mismatch repair (MMR) [10, 11, 20, 24]. These examples underline how genetic profiles of RCC can direct precision medicine [25–27] and improve clinical outcome.

However, at the state of art, there is no molecular signature capable to accurately predict clinical outcome of RCCs. The research of specific molecular characteristics of RCC is of primary importance for the management of this malignancy.

In this case report, we describe an unusually genomic instable case of renal cell cancer characterized by predisposing germline mutations in *BRCA1* and *RAD51* genes. We detected increase of mutational rate, microsatellite instability (MSI) and alterations in genes related to DNA repair in the tumour genome that may benefit the cancer response to the immunotherapy.

## Results and discussion

Here, we report the case of a 65-years-old male patient, part of our background cohort of 365 RCC. In October 2020, the patient, previously asymptomatic, received the diagnosis of primary malignant neoplasm of kidney. According to the histopathological investigation the tumour was classified as moderately differentiated RCC (G2). Neoplastic lesions showed kidney vasculature and peripelvic fat invasion (stage III). At the time of diagnosis, no tumor spread to regional lymph nodes and no involvement of distant organs was detected. TNM classification was pT3a cN0/cM0 L0 V1 R0.

The patient underwent complete surgical tumor resection without need of following adjuvant therapy. In February 2021 during the follow-up, the patient was found to have a suspicious nodule for metastases at the level of the right diaphragmatic peritoneum, identified by a computed tomography scan. In May 2021, the patient displayed a high level of prostate-specific antigen (PSA) and a suspicious prostate nodule. Afterwards, no further data on patient follow-up are available. The anamnesis indicated no familial history of RCC. The patient was not a smoker and not affected by obesity.

**Fig. 1:**
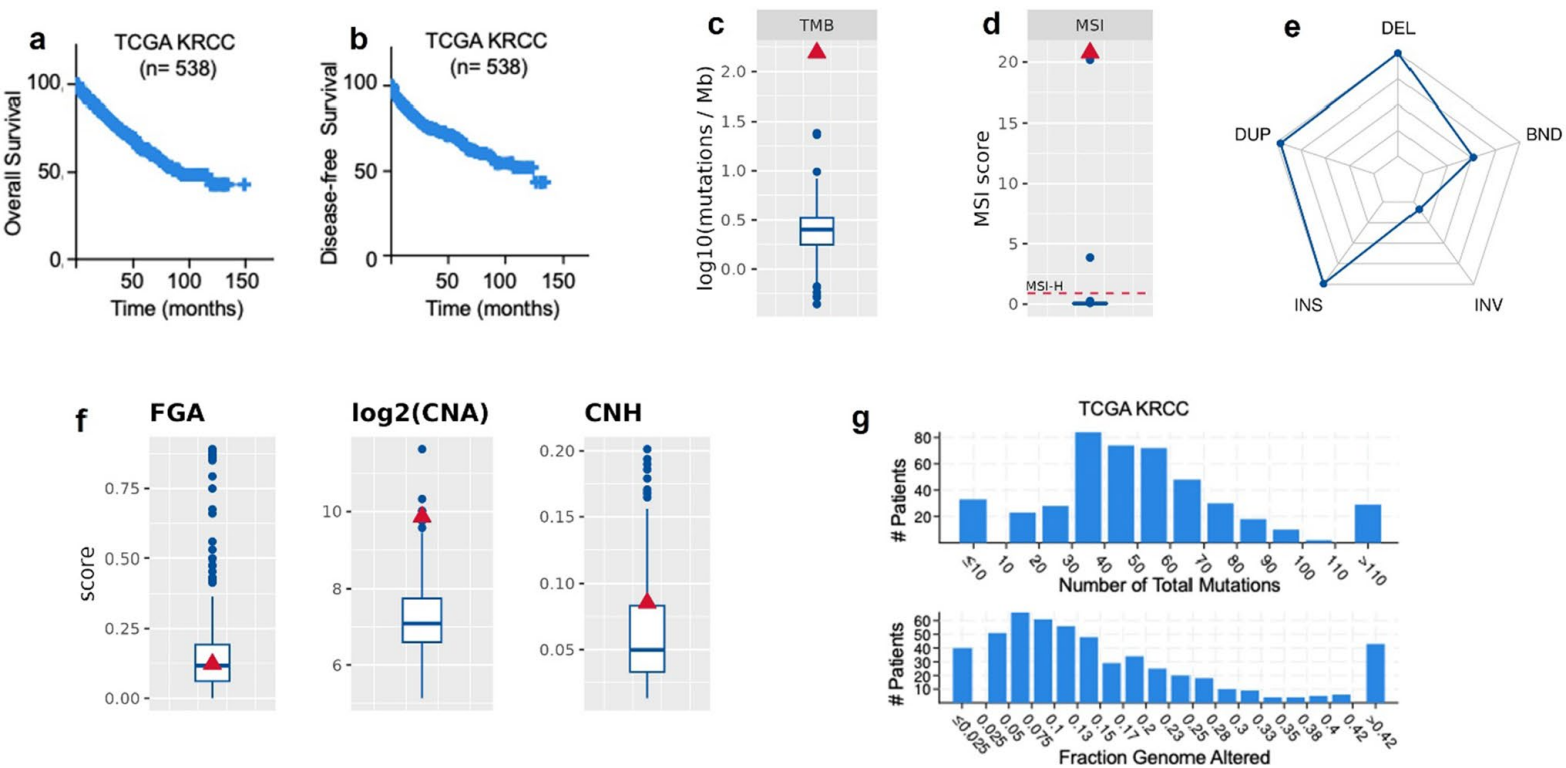
Genomic instability in the patient is represented by multiple metrics: **a**, **b** overall survival (OS) of RCC estimates by Analysis on the Cancer Genome Atlas (TCGA), **c** high tumor mutational burden, **d** MSI-H status, **e** much more deletion, insertions, duplications and break ends than average RCCs, **f** highly structural instable (CNA), intra-tumor heterogeneity slightly increased (CNH), but average numerical CIN score (FGA). The patient (red triangle) is compared to the clinical cohort (blue boxplot). **g** Graphs show total mutations and fraction genome altered in RCC

Analysis on the Cancer Genome Atlas (TCGA) estimates the overall survival (OS) of RCC of approximately 60% at 5 years from diagnosis, correlated with a significant incidence of tumor relapse within 10 years (Fig. 1a, b). We conducted a multi-omics analysis to identify biomarkers that can predict response to specific targeted therapies.

Whole genome sequencing analysis of the patient’s tumor detected several somatic mutations in cancer-related genes (Table 2).

**Table 2:** Mutation in therapy related target genes detected in the patient

Gene	Position	Original AA	Alteration	VAF (%)
EGFR*,^_^	511	Ser	Tyr	52.40
EML4*	398	Lys	Arg	50
ERBB2*,^*_*^	8	Pro	Thr	54.50
*CSF3R°*	835	Glu	Lys	64.20
*EGFR°*,^*_*^	511	Ser	Tyr	52.40
*EPHB2°*	750	Arg	Cys	42.60
*ERBB2°*,^*_*^	8	Pro	Thr	54.50
*FLT4°*	1146	Arg	His	30.60
*PIK3CB°*	475	Pro	Ser	50.50
*POLD1°*	875	Arg	His	49.50
*FLT4*	1146	Arg	His	30.60
*PIK3CB*	475	Pro	Ser	50.50
*POLD1*	875	Arg	His	49.50
*AR*	473	Gly	duplication	96.30
*ATM*	1853	Asp	Asn	46.40
*ATRX*	929	Glu	Gln	100
*ATXN7*	264	Lys	Arg	69.30
*BRCA2*	372	Asn	His	100
*CASP8*	344	Asp	His	46
*CRLF2*	323	Ser	Phe	45.90
*CYSLTR2*	201	Met	Val	51.50
*ERCC2*	312	Asp	Asn	48.60
*ETV1*	100	Ser	Gly	46.20
*FCGR2A*	63	Gln	Trp	51.50
*FOXP1*	202	Gln	His	36.20
*GSTP1*	105	Ile	Val	100
*HLA-C*	327	Val	Met	51.20
*IL7R*	244	Thr	Ile	45.10
*IRS2*	1057	Gly	Asp	45.90
*JARID2*	492	Arg	Cys	52.40
*KMT2A*	30	Ala	Gly	45.70
*MSH3*	60–62	-	deletion	40.20
*MYC*	79	Gly	Cys	52.10
*NOTCH3*	817	Pro	Leu	55.10
*NRG1*	286	Met	Thr	51.70
*PARP1*	123	Lys	Arg	52.10
*PBRM1*	1584	Pro	frameshift	10.30
*PRKAR1A*	333	Ser	Asn	58.50
*PTCH1*	1164	Pro	Leu	46
*RAD23B*	249	Ala	Val	47.70
*SERPINB3*	357	Thr	Ala	55.10
*TET2*	1783	Ile	Val	46.10
*TP53*	384	Ile	Phe	42.20
*VHL*	148	Phe	frameshift	34.80
*WWTR1*	74	Pro	Gln	47
*MSH5*			missense	

° Somatic mutations detected in the patient by whole genome sequencing. * Off-label ° Therapy targeting gene is in clinical trials. ^**_**^: Therapy targeting gene is FDA approved in another disease, but it is also in clinical trials in the patients’ disease. * Off-label = Therapy targeting gene is FDA approved only in another disease. *VAF* Variant allele frequency

Unfortunately, no FDA-approved drugs are available for this mutational profile. Among gene alterations, we identified mutations in CSF3 (Colony stimulating factor 3), EGFR (Epidermal growth factor receptor), EPHB2 (EPH receptor B2), ERBB2 (Erb-b2 receptor tyrosine kinase 2), FLT4 (Fms related receptor tyrosine kinase 4), PIK3CB (Phosphatidylinositol-4,5-bisphosphate 3-kinase catalytic subunit beta), POLD1 (DNA polymerase delta 1, catalytic subunit) genes for which therapies targeting gene are in clinical trials.

The patient displays also somatic mutations in *TP53* (tumor suppressor protein 53), clearly involved in cancer biology [28–31] and in key genes of DNA repair pathway as *BRACA2* (BRCA2 DNA repair associated), *MSH3* (MutS homolog 3) and *MSH5* (MutS homolog 5). Remarkably, the patient displayed a highly genomic instable renal cell cancer, as shown by a high mutational burden (Fig. 1c), and high microsatellite instability (MSI-H, Fig. 1d) compared to the average of the 365 patients in Indivumed’s RCC cohort. We also detected an unusually high frequency of chromosomal aberrations as deletions, duplications, insertions and breakends (Fig. 1e) compared to the average of RCC cases, another feature of genome instability. TCGA analysis confirmed that generally renal cell carcinoma genome is characterized by low mutational rate and low percentage of genome affected by copy number variations (CNV) (Fig. 1f, g).

**Fig. 2:**
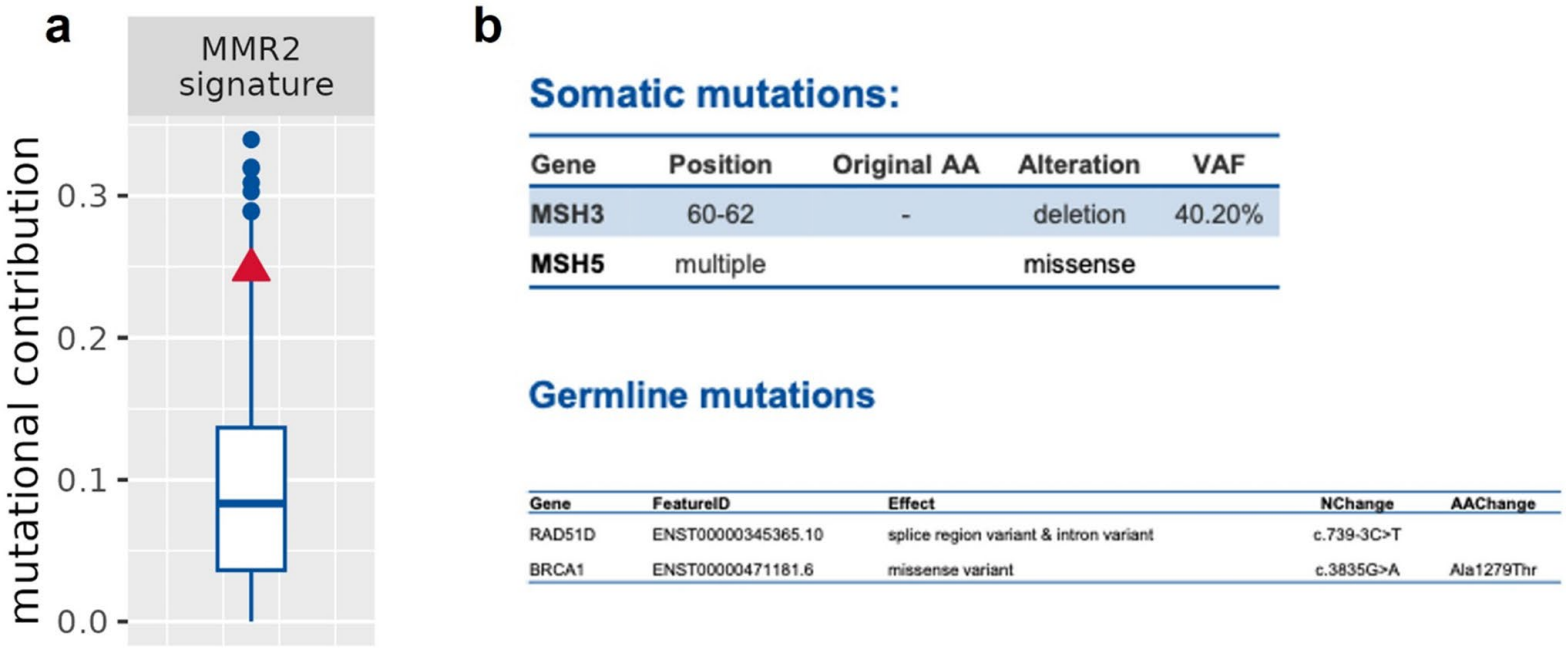
MMR signature. **a** Mutational contribution of the mismatch repair related signature. The patient (red triangle) is compared to the clinical cohort (blue boxplot). **b** The main somatic and germline mutations

We next performed a whole cancer genome sequencing of the patient's tumor tissue. This reported an enrichment of the MMR2 mutational signature not common to RCC (Fig. 2a). The MMR2 mutational signature is associated to defective DNA mismatch repair (MMR) and inactivation of genes involved in this DNA repair mechanism. Accordingly, the patient showed among others, deletion of 3 nucleotides in *MSH3* gene and multiple missense mutations in *MSH5* gene, both involved in DNA mismatch repair (Fig. 2b). Notably, MMR system deficiency has been associated to an increase of mutation burden [32]. Additionally, MMR-associated mutational signatures have been reported enriched in colorectal and gastric adenocarcinomas with high microsatellite instability [33].

Analysis of adjacent, non-cancer derived DNA from the patient allowed identification of germline mutations in *RAD51D* (c.739-3C>T) and *BRCA1* (c.3835G>A) genes (Fig. 2b, lower panel).

Products of both genes, *RAD51D* and *BRCA1*, are involved in homologous recombination (HR), the high-fidelity repair pathway for DNA double strand break (DSB). Loss of RAD51 paralog, RAD51D, leads to HR deficiency and triggers deletion of chromosome segments located close to DSB site caused by excessive end-resection [34] while BRCA1 ensures genome integrity by regulating cell cycle checkpoints and DNA repair [35]. RAD51D and BRCA1 germline mutations are causative of genetic predisposition to develop ovarian and breast cancer [35–38] and their clinical relevance in RCC is still unknown.

Only few cases have described BRCA1 germline mutations in patients affected by renal cell carcinoma. In 2011, the 2080insA BRCA1 germline mutation was described for the first time in 45-year-old Pakistani patient affected by aggressive form of clear cell renal carcinoma [39]. Germline mutations in DDR-related genes, among them BRCA1, have been also reported in RCC patients in Chinese (0.6% cases) and Polish (0.4% cases) population, respectively [40–42]. BRCA1 and RAD51D germline mutations may therefore underlie predisposition to RCC and might have cooperated in our patient determining this unusual high genome instability profile. Nonetheless, since the BRCA1 germline mutations has been detected in distinct cancers, sometimes without clear pathological significance [43–46], further investigations are required to determine the impact of BRCA1 germline mutations on RCC.

**Fig. 3:**
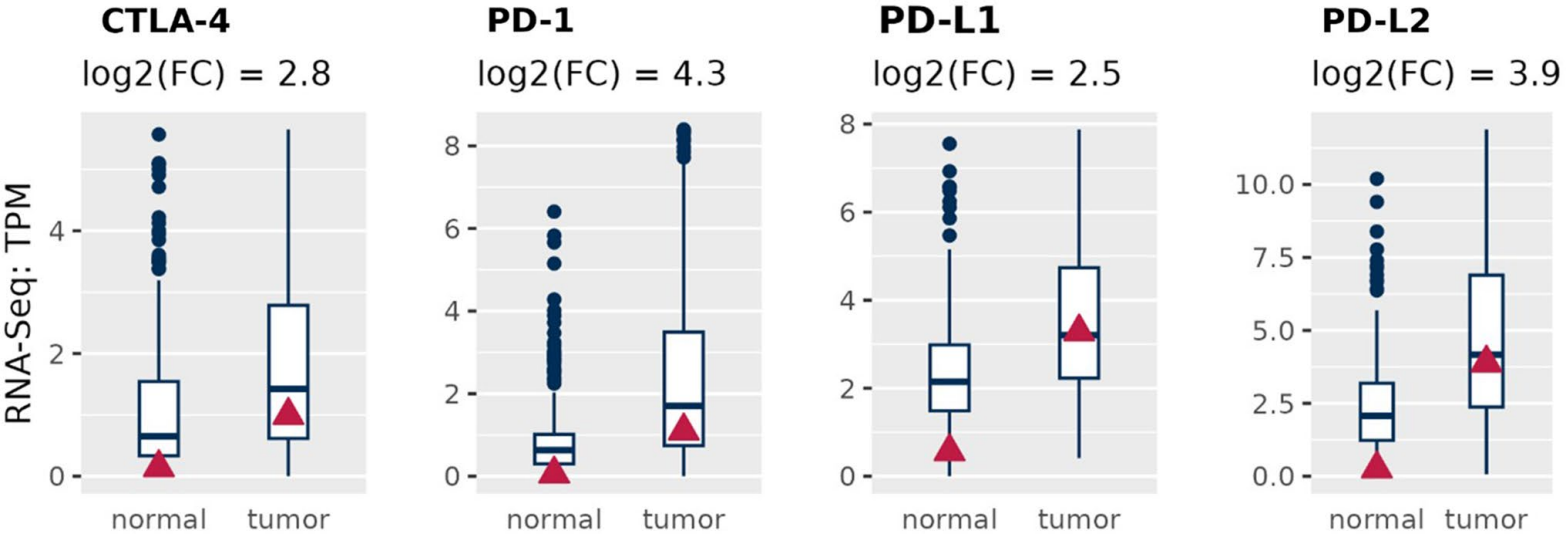
RNA-Seq expression levels of immune checkpoint genes in the patient. The patient (red triangle) is compared to the clinical cohort (blue boxplot)

The enrichment of MMR-2 signature confers hypersensitivity to immunotherapy, somatic mutations in *EGFR* and *HER2* genes are associated, respectively, to tumor response to EGFR tyrosine kinase inhibitors (TKI) and a better outcome in response to immune checkpoints inhibitor therapy (anti PDL-1 and anti CTL4) [47, 48]. Based on this evidence, we analyzed the tumor expression of the immune checkpoints PD-1, PDL-1, PD-L2 and CTL4 which are notably involved in sustaining self-tolerance in tumor site (PD-1/PDL-1) and in the lymph node (CD28/ CTL-4) by modulating the immune response. Elevated expression of them suggests a possible strategy of cancer to mask itself and to escape from immune surveillance. Indeed CD28/ CTL-4 signaling pathway enhances immunosuppression supported by Tregs while PD-1/PDL-1–2 overexpression by cancer cells leads to the inhibition of T-cell activity that confers tumor immune resistance [49]. We found a global up-regulation of PD-1, PDL-1, PD-L2 and CTL4 in this investigated RCC compared to the normal controls (Fig. 3).

Overall, up-regulation of immune checkpoints [24, 50, 51], as well as high tumor mutational burden and elevated microsatellite instability [50–55] (Fig. 1) are criteria that may predict tumor susceptibility to the immunotherapy. Thus in 2021, the patient here reported was included in a randomized controlled trial using a combination of immunotherapeutic agents, Nivolumab (anti-PD-L1) and Ipilimumab (anti-CTL4).

In conclusion, the high genome instability found in this isolate case of RCC may confer tumor hypersensitivity to immunotherapy, the most prominent therapeutic approach for RCCs. In addition, within the framework of personalized medicine [56–59], the here described unusual somatic mutations could provide great opportunities capable of improving the management of RCC patients.

## Material and methods

### Collection of samples

Tumor tissues were globally collected using a standardized protocol, minimizing the ischemia time until freezing in liquid nitrogen [60–62]. To ensure the quality of the samples, all tissues were Hematoxylin and Eosin stained [63, 64] and subjected to a pathological QC as previously described [65]. Approximately 10 mg tissue were taken for nucleic acid extraction and protein lysate preparation each.

### Nucleic acid extraction and quality assessment

Frozen tissue slices were mixed with beta-mercaptoethanol containing sample buffer and homogenized using the BeadBug system [66, 67]. DNA and RNA were extracted in parallel from the same sample using the Qiagen AllPrep Universal Kit according to the manufacturer’s instructions, as well as using biochemical methods [68, 69].

DNA and RNA concentration were quantified using Qubit fluorometer with the Qubit dsDNA BR assay or Qubit RNA BR assay respectively.

DNA and RNA quality were assessed using the Agilent Tapestation with the Agilent Genomic DNA kit or Agilent High-Sensitivity RNA ScreenTape kit respectively. RNAs need to have a RIN ≥ 4 or a DV200 ≥ 60 to be selected for library preparation.

### Library preparation and NGS sequencing

Libraries for whole genome sequencing (WGS) were performed as recently described by Yang et al. [70].

### NGS data processing

NGS data was aligned against Grch38 genome assembly. Haplotype Caller (genome analysis toolkit; GATK) [71] was used for short genomic identification and annotation in normal sample. The following consensus ere used for WGS somatic variations: Mutect2 [72], Strelka [73], Varscan [74] and Somatic Sniper [75]. Structural variations were called using R packages TitanCNA [76], DellyCNV and DellyCall [77], as well as Manta [78].

RNA-Seq differential expression was based on normalized readcount data (TPM: transcripts per million).

### Bioinformatical analyses

R package MutationalPatterns [79–81] was used for mutational signatures calculation whilst R package MSIseq [82] was used for MSI classification. Metrices to define chromosomal instability were determined using R package CINmetrics [83] and CNHplus [84].

Aneuploidy events were analysed using ASCETS [85]. Aneuploidy event span more than 90% of the chromosome. Visualization of results was done in IGV [86].

TMB was calculated as the number of non-synonymous mutations of protein coding genes divided by exome size in Megabases.

## Data Availability

The datasets generated and/or analyzed during the current study are available from the corresponding author on reasonable request.
